# MRI evaluation of right and left ventricular remodeling and surrogate markers of PH following acute pulmonary embolism

**DOI:** 10.1186/1532-429X-11-S1-O59

**Published:** 2009-01-28

**Authors:** Frederikus A Klok, Soha AH Romeih, Jos JM Westenberg, Lucia JM Kroft, Menno V Huisman, Albert de Roos

**Affiliations:** LUMC, Leiden, Netherlands

**Keywords:** Pulmonary Embolism, Pulmonary Hypertension, Relative Decrease, Ventricular Remodel, Acute Pulmonary Embolism

## Introduction

It is difficult to identify patients at risk for chronic-thromboembolic pulmonary hypertension (CTEPH) in the clinical course of pulmonary embolism (PE). We evaluated ventricular remodeling and markers of pulmonary hypertension (PH) using cardiac MRI in PE patients after 6 months treatment.

## Methods

Fifteen PE-patients and 10 controls, in whom PE was suspected but ruled out, were studied. A baseline CT scan was performed to diagnose PE and to assess dynamic right and left ventricular (RV; LV) function. After 200 days, a MRI scan was performed to assess bilateral ventricular function and several surrogate markers of PH.

## Results

Baseline characteristics of both controls and PE-patients were comparable. In the control cohort, end-systolic (ESV) and end-diastolic volume (EDV) in both ventricles did not change in time. PE patients with normal RV function at baseline had a significant improvement in RVEF (+5.4 ± 3.1%) due to a relative decrease in ESV (-17 ± 7.9%). Patients with abnormal RV function at baseline had a significant improvement in RVEF (+14 ± 15%) due to relative decrease in both ESV (-36 ± 23%) and EDV (-22 ± 16%). Furthermore, LVEDV increased significantly (15 ± 11%).

Pulmonary distensibility index (0.033 ± 0.0058) was significant decreased in patients with persistent RV dysfunction compared to patients with restored RV function (0.22 ± 0.18) and controls (0.28 ± 0.25). In addition, decreased stroke volume (71 ± 21 ml versus 103 ± 40 ml and 94 ± 19 ml respectively) and PH specific alterations in the pulmonary flow curves were found in these patients compared to both other groups.

## Conclusion

RV remodelling after PE is dependent on the degree of baseline RV dysfunction. RVEF improves due to decrease in RVESV and in lesser extent to RVEDV. Compared to controls and PE patients with normalized RV function, patients with persistent RV dysfunction have PH specific pulmonary flow alterations and a stiffened pulmonary artery. It remains to be studied whether these patients are at risk for developing CTEPH.

